# Deposition Rate‐Controlled Functional Evolution of p‐Type Selenium‐Alloyed Tellurium–Tellurium Oxide Films for Thin‐Film Transistors

**DOI:** 10.1002/advs.77618

**Published:** 2026-09-08

**Authors:** Seongmo Kang, Jeong‐Min Seo, Hyungyu Choi, Yunseo Song, Tae Woong Yoon, Tae Woong Han, Hoseong Shin, Jiyun Lee, Boseok Kang

**Affiliations:** ^1^ SKKU Advanced Institute of Nanotechnology (SAINT) and Department of Nano Science and Technology Sungkyunkwan University Suwon Gyeonggi‐do South Korea; ^2^ Department of Nano Engineering and Department of Semiconductor Convergence Engineering Sungkyunkwan University Suwon Gyeonggi‐do South Korea

**Keywords:** deposition rate, p‐type oxide semiconductors, tellurium oxide, thermal evaporation, thin‐film transistors

## Abstract

The development of p‐type amorphous oxide semiconductors (AOSs) has long been a major challenge that limits the advancement of oxide electronics. Selenium‐alloyed tellurium−tellurium oxide (SeTe–TeO_x_) has emerged as a promising p‐type AOS with electrical properties and stability comparable to n‐type counterparts. However, the influence of deposition kinetics on the functional properties remains unexplored. Here, the thermal deposition rate is systematically controlled to investigate its impact on the chemical structure, microstructure, and optoelectronic properties of SeTe–TeO_x_ films. Thermodynamic studies and experimental analyses reveal that increasing the deposition rate promotes the formation of Te–Te metallic bonds within the oxide matrix, enabling effective modulation of electrical properties while maintaining a near‐amorphous structure. Thin‐film transistors exhibit an enhancement in average field‐effect mobility from 8.2 to 20.0 cm^2^ V^−1^ s^−1^ along with improved bias stability. This tunability reflects a transition from percolation‐limited to well‐percolated transport with increasing deposition rate. Complementary analyses further reveal that the well‐connected transport network suppresses the macroscopic influence of kinetically slow trapping, despite a greater contribution from interfacial and near‐band‐edge states. These findings provide fundamental insights into the process–property linkages in SeTe–TeO_x_ and establish deposition rate as a simple yet powerful strategy for engineering p‐type oxide electronics.

## Graphical abstract

This study investigates the effects of thermal deposition rate on next‐generation p‐type amorphous oxide semiconductor SeTe–TeO_x_ films. Deposition rate governs the chemical states, microstructure, and electrical characteristics of the films, resulting in tunable device properties. These findings provide a practical pathway for the rational design and optimization of p‐type amorphous oxide semiconductors for future electronics.

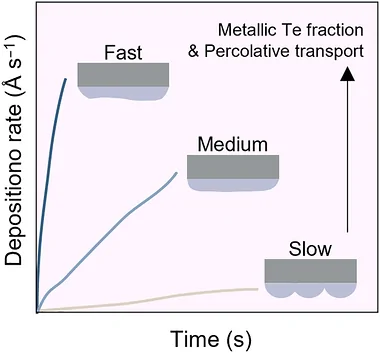

## 1 Introduction

Since the successful commercialization of indium‐gallium‐zinc oxide, amorphous oxide semiconductors (AOSs) have emerged as compelling channel materials for the display industry due to their high electron mobility, low process temperature, and excellent large‐area uniformity [1, 2, 3, 4, 5, 6, 7, 8]. These advantages stem from the ionic bonding character between heavy post‐transition metal cations and oxygen anions [2, 9]. The large electronegativity difference drives partial electron transfer from metal *n*s orbitals to oxygen 2p orbitals, forming strong electrostatic interactions. As a result, spatially extended and isotropic metal *n*s orbitals predominantly constitute the conduction band minimum (CBM), enabling effective orbital overlap and efficient electron transport even in amorphous structures. In contrast, the valence band maximum (VBM) is composed of localized and anisotropic oxygen 2p orbitals. This leads to a large effective hole mass, a high density of oxygen‐related defect states near the VBM, and strong compensation of native acceptors [10, 11, 12]. Such intrinsic limitations make it fundamentally challenging to realize highly hole‐conductive and stable p‐type characteristics. Therefore, the absence of high‐performance p‐type AOSs remains a critical bottleneck for next‐generation oxide electronics, including all‐oxide complementary metal‐oxide‐semiconductor (CMOS) technology [13, 14].

To address this challenge, various material design strategies have been proposed to mitigate the VBM localization. Among them, the most extensively investigated approach is hybridizing oxygen 2p orbitals with metal orbitals of comparable energy levels. Copper oxides, tin oxide, and nickel oxide are representative p‐type oxide semiconductors adopting this concept. However, these materials still suffer from low hole mobility and poor environmental stability even under optimized conditions [15, 16, 17]. Recently, Liu et al. reported selenium‐alloyed tellurium–tellurium oxide (SeTe–TeO_x_)‐based thin‐film transistors (TFTs) that exhibit a field‐effect mobility (*µ*_FE_) of ∼15 cm^2^ V^−1^ s^−1^, highlighting their potential compatibility with CMOS integration [18]. The authors employed thermal evaporation of Se and TeO_2_ mixed powder, during which partial decomposition of TeO_2_ generates elemental Te. This leads to the formation of a percolated network of Te–Te metallic bonds dispersed within an amorphous TeO_x_ matrix. Within this structure, delocalized Te 5p states near the VBM provide shallow acceptor states and enable efficient hole transport. In addition, Se incorporation suppresses excessive hole concentration and improves orbital connectivity. Consequently, this multi‐component hybrid system achieves high *µ*_FE_, reduced off‐current (*I*_off_), and improved subthreshold swing (*SS*) with robust operational stability.

Following the emergence of SeTe–TeO_x_ as a promising p‐type AOS, research has expanded into its charge transport and defect physics [19, 20, 21], circuit integration [22, 23], and diverse device applications [24, 25, 26, 27, 28]. Among these, several studies have examined property evolution dictated by compositional variation [22, 23]. However, the role of deposition rate in governing the formation of SeTe–TeO_x_ films remains largely unexplored. In thermal evaporation, the deposition rate is closely coupled to the source temperature. Even subtle changes in deposition rate can therefore drastically alter the dominant vapor species and the resulting chemical structure. This effect becomes particularly important in multi‐component systems such as SeTe–TeO_x_, where the evaporation process involves complex thermodynamic behavior of the constituent materials [29, 30, 31, 32, 33]. The deposition rate also governs the growth kinetics and atomic network densification, which directly determine the charge transport characteristics. Therefore, a systematic investigation into the deposition rate‐driven functional evolution is essential to deepen our understanding of this novel material.

In this article, we establish a rate‐dependent deposition mechanism by systematically controlling the thermal deposition rate, linking the resulting microstructure, chemical structure, and electrical evolution of SeTe–TeO_x_ films. We demonstrate that increasing the deposition rate intensifies thermal decomposition of TeO_2_, shifting the vapor flux from a Te–O‐containing to an elemental Te‐enriched phase. The change in dominant vapor species, combined with the reduced inter‐arrival time of adatoms, directly modulates the charge transport characteristics. TFTs fabricated with these films exhibit an increase in average *µ*_FE_ from 8.2 to 20.0 cm^2^ V^−1^ s^−1^, accompanied by a reduction in hysteresis (*ΔV*_th_). We further probe the charge trapping landscape and explore additional optimization through film thickness variation. This work provides a comprehensive process–property study of SeTe–TeO_x_ and offers practical guidelines for the rational design of next‐generation p‐type oxide electronics.

## 2 Results and Discussion

### 2.1 Rate‐Controlled Thermal Evaporation

**FIGURE 1:**
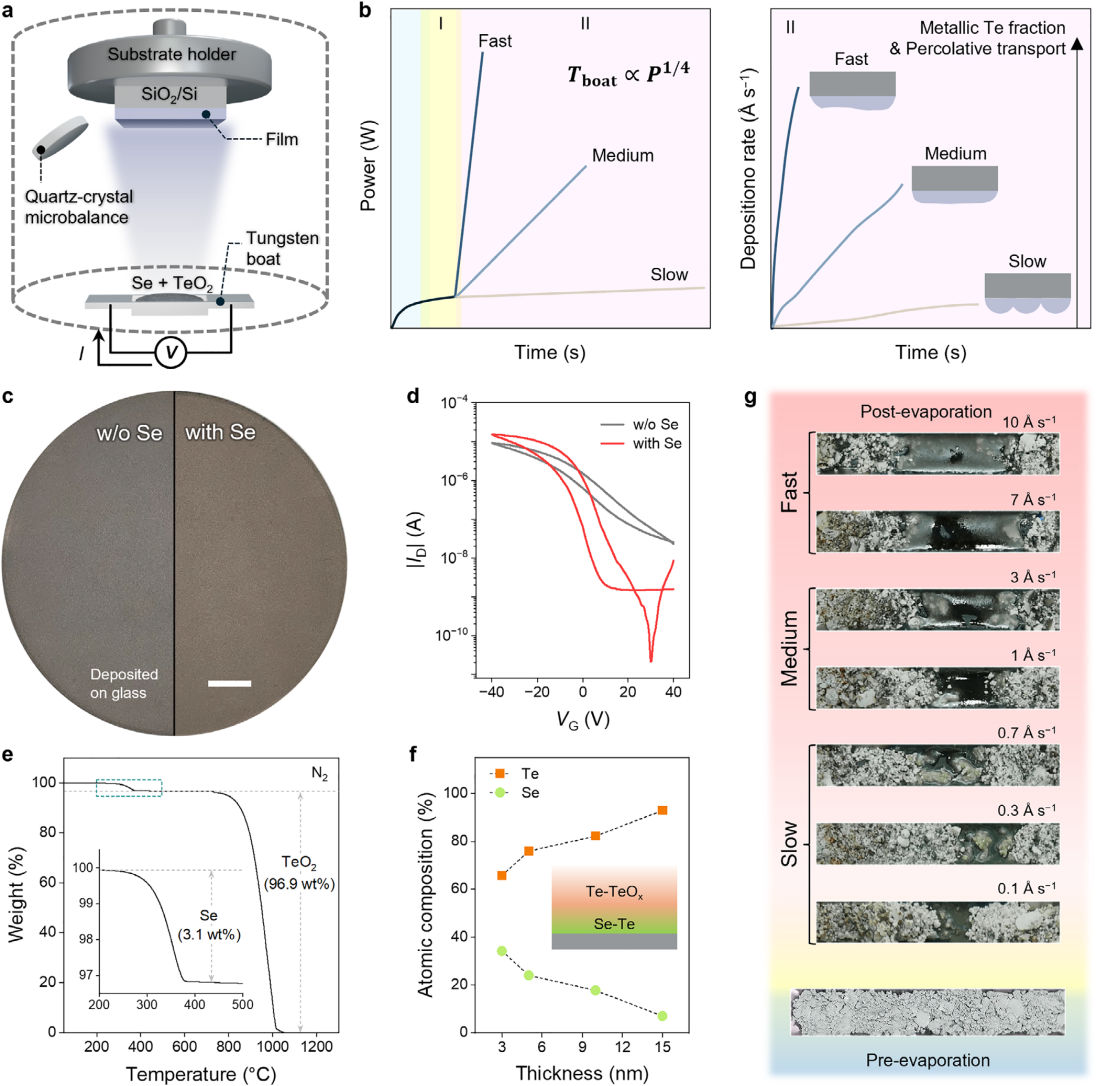
Rate‐controlled deposition of SeTe–TeOx films. (a) Schematic illustration of the thermal evaporation system. (b) Supplied electrical power (left) and the corresponding deposition rate (right) as a function of time. (c) Photograph of Te–TeO_x_ films deposited on glass without (left half) and with (right half) Se (scale bar, 5 mm). (d) Transfer characteristics of TFTs fabricated with and without Se. (e) TGA curve of the Se/TeO_2_ mixed source; the inset shows an enlarged view of the 200–500 °C region (dashed box). (f) Atomic composition of Se and Te as a function of film thickness, measured by XPS on 3, 5, 10, and 15 nm films. (g) Photographs of pre‐ and post‐evaporation boats after deposition at 0.1, 0.3, 0.7, 1, 3, 7, and 10 Å s^−1^.

Figure 1a illustrates the rate‐controlled thermal evaporation system. A mixture of 12 mg Se and 400 mg TeO_2_ powder was loaded into a tungsten boat. In this setup, we control the electrical power delivered to the boat while monitoring the deposition rate and film thickness using a quartz‐crystal microbalance (QCM). The input power determines the heat supplied to the source, which governs the vapor pressure of the source material and the flux of adatoms arriving at the substrate, that is, the deposition rate (a detailed explanation is provided in Note S1). To systematically examine the influence of deposition rate on the structural, chemical, and electrical properties of 15‐nm‐thick SeTe–TeO_x_ films, a two‐stage power ramp was employed. In the first stage, the power was ramped at a fixed speed to a pre‐deposition threshold (Figure 1b(I)). Thereafter, the power was linearly ramped at different speeds to reach seven target rates, 0.1, 0.3, 0.7, 1, 3, 7, and 10 Å s^−1^ (Figure 1b(II)), providing sufficient resolution to capture the transitions among different deposition rate regimes over the experimentally accessible range. This approach enables precise control of both the accessible temperature range and the residence time of the source at specific temperatures without provoking power overshoot, source spitting, or discontinuous film growth. The actual film thicknesses were verified by atomic force microscopy (AFM), and the QCM tooling factor was individually calibrated for each deposition rate to maintain comparable values across all conditions (Figures S1 and S2).

Previous studies have reported that Se incorporation effectively modifies the electronic structure of Te–TeO_x_ films by suppressing excessive metallic Te–Te states and compensating for oxygen vacancies, thereby improving p‐type conductivity [22, 34, 35]. Consistent with these reports, the Se‐alloyed film deposited on glass appears lighter in color than the film deposited without Se (Figure 1c; original photograph in Figure S3), reflecting the reduced visible‐light absorption and the increase in the optical bandgap ($E_{g}^{\mathrm{opt}}$). The Se incorporation also improves the transfer characteristics of the TFTs (Figure 1d), as manifested by an enhanced on‐current (*I*_on_), a substantially reduced *I*_off_, and a lower *SS*.

To understand the thermal behavior of the mixed Se/TeO_2_ source, we conducted thermogravimetric analysis (TGA) under N_2_ flow (Figure 1e). Two distinct weight losses were observed at ∼220 °C and ∼730 °C, which correspond to the melting points of Se and TeO_2_, respectively [36, 37]. The first weight loss of ∼3.1% is attributed to Se volatilization and closely matches the nominal Se fraction in the source (1/33 ≈ 3.03%). Before the second drop, differential scanning calorimetry (DSC) exhibited a broad endothermic feature followed by a sharp exothermic event at ∼730 °C (Figure S4a), suggesting that melting and partial decomposition or restructuring may occur prior to bulk TeO_2_ evaporation [38]. These results indicate that Se and TeO_2_ follow their individual thermal behaviors even within the mixture: Se depletes at a lower temperature, while TeO_2_ persists to higher temperatures, undergoing complex reactions. The TGA of the post‐evaporation remnant supports this interpretation, showing negligible weight loss between 220 °C and 400 °C (Figure S4b).

To verify that the above conclusion holds under actual deposition conditions, we prepared films with thicknesses of 3, 5, 10, and 15 nm and quantified the Se and Te atomic composition using X‐ray photoelectron spectroscopy (XPS) (Figure S5). Ion‐etching XPS depth profiling was intentionally avoided to prevent film damage and potential signal distortion [39]. As shown in Figure 1f, the Se fraction is highest in the 3 nm film and decreases monotonically with increasing thickness, consistent with the cross‐sectional energy‐dispersive X‐ray spectroscopy analysis presented in Figure S6. These results support the preferential volatilization of Se during the initial stage of deposition [35]. Notably, the early presence of Te suggests that TeO_2_ and its partially reduced suboxides can vaporize even at relatively low temperatures. This behavior is plausible given that the source is heated under a reducing environment during deposition. The high vacuum (∼10^−6^ Torr) and the resulting low oxygen partial pressure ($p_{O_{2}}$) facilitate oxygen loss at the source surface, while the tungsten boat, a highly refractory metal, may further promote the decomposition of TeO_2_ at the contact interface [40]. Under these conditions, the formation of suboxides becomes thermodynamically favorable, as quantitatively analyzed in Note S2.

In Figure 1g, photographs of the pre‐ and post‐evaporation boats provide visual insight into evaporation kinetics (original images in Figure S7). Up to 0.7 Å s^−1^, the residual powder appears predominantly scorched, whereas a glossy and fused region becomes apparent at 1 Å s^−1^ and above. Based on this, we infer that active melting occurs near the temperature corresponding to 1 Å s^−1^. In addition, the pronounced darkening of the residue suggests the presence of elemental Te, which may arise from the disproportionation of TeO (e.g., 2TeO → TeO_2_ + Te) [40, 41]. These observations corroborate that the deposition process involves a complex, noncongruent vaporization pathway of TeO_2_ and its reduced species [36, 41, 42].

### 2.2 Rate‐Dependent Deposition Mechanism and Microstructural Analysis

**FIGURE 2:**
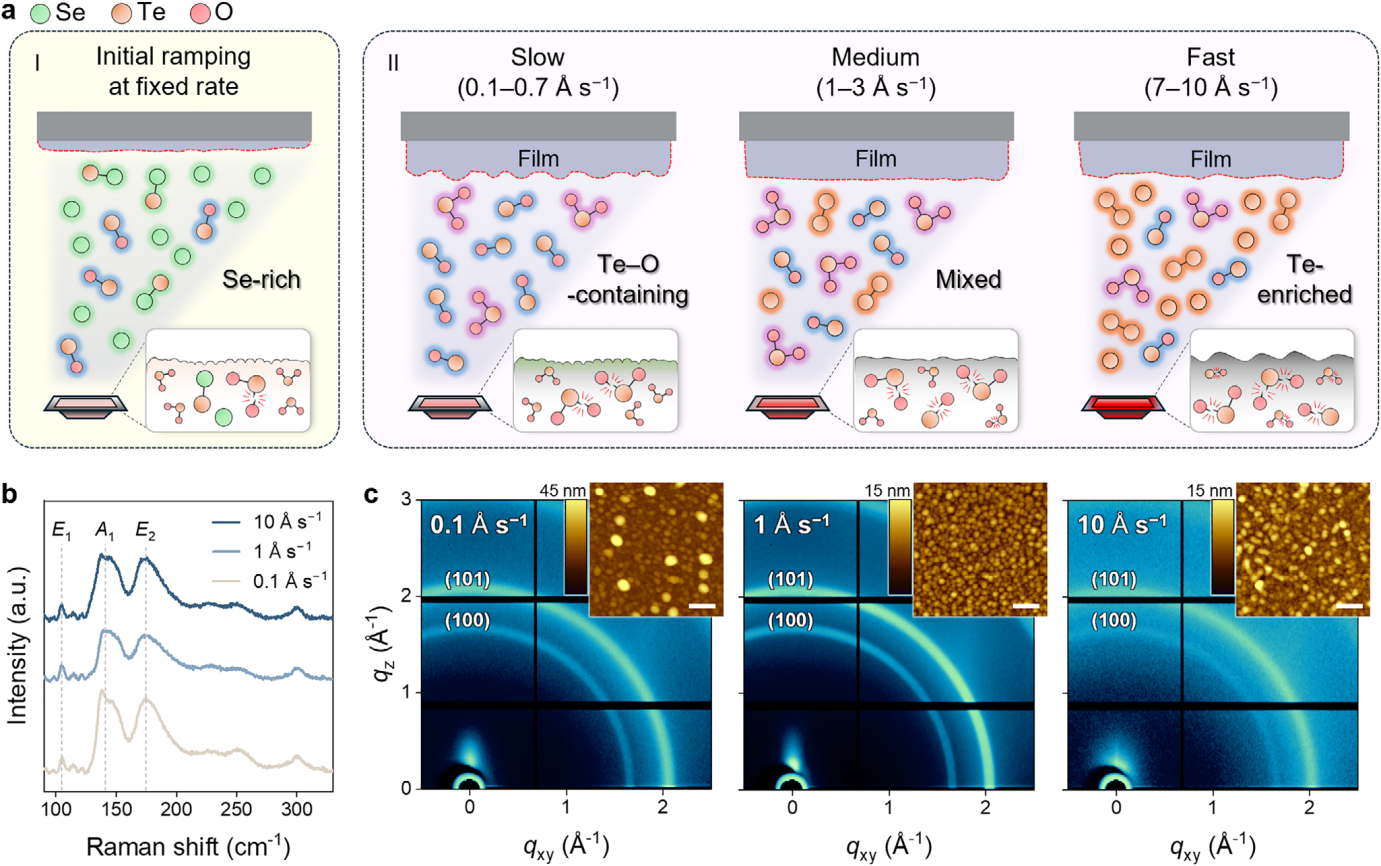
Rate‐dependent deposition mechanism and microstructural analysis. (a) Schematic illustration of the proposed deposition mechanism, describing the thermodynamic behavior of the source, the resulting vapor flux composition, and the film growth kinetics during (I) the initial ramping stage and (II) the subsequent slow, medium, and fast regimes. (b) Raman spectra and (c) 2D GIWAXS images with corresponding AFM topography insets for the films deposited at 0.1, 1, and 10 Å s^−1^. Scale bar, 0.2 µm.

Guided by experimental evidence (Figure 1f–g) and the thermodynamic study of TeO_2_ (Note S2), we propose a rate‐dependent deposition mechanism that describes the major vapor species and microstructural evolution of the films (Figure 2a). As discussed in Section 2.1, the initial power ramping stage produces Se‐rich vapor, accompanied by the vaporization of partially reduced suboxide species (Figure 2a(I)). Subsequently, TeO_2_ undergoes complex thermal decomposition and vaporization processes (Figure 2a(II)). For simplicity, we limit our scope to the major Te–O species: TeO_2_, TeO, and elemental Te (Te_2_) [36], which are highlighted with purple, blue, and orange backgrounds, respectively. Based on the comprehensive analysis that follows, the seven rates are categorized into slow (0.1–0.7 Å s^−1^), medium (1–3 Å s^−1^), and fast (7–10 Å s^−1^) regimes.

In the slow regime, the limited thermal energy suppresses the extensive melting and evaporation of TeO_2_. Within this temperature window, thermodynamic analysis suggests that the reducing environment renders dissociative vaporization pathways, such as TeO_2_(s) → TeO(g) + ½O_2_(g), relatively favorable compared with congruent sublimation, increasing the contribution of reduced Te–O species to the vapor flux. Upon condensation, these reduced Te–O species may not remain stable as discrete condensed phases and may instead undergo disproportionation or oxidation by residual oxygen, thereby promoting the recovery of Te–O bonds [36, 41]. Moreover, the low flux density reduces gas‐phase collisions and prolongs the surface residence time before burial by subsequent arrivals, enabling extensive surface diffusion [43]. Consequently, surface energy‐driven aggregation dominates, and the film evolves into large, densely coalesced island‐like clusters [29, 44].

In the medium regime, increased thermal input initiates active melting and effectively lowers the barrier for vaporizing molecular TeO_2_. As the melt becomes fluid, the formation of a reduced surface layer is constantly suppressed, and the local oxygen potential near the source increases. Consequently, congruent evaporation of TeO_2_ becomes more significant, leading the vapor composition to approach a dynamic equilibrium between TeO_2_ and the reduced suboxide species (a mixed vapor flux) [36, 42]. Meanwhile, the higher arrival rate increases gas‐phase collisions and shortens the burial time. This allows subsequent adatoms to be incorporated into the growing front before excessive surface diffusion or coalescence occurs, thereby inhibiting re‐oxidation and excessive surface energy‐driven clustering. As a result, the film grows with a more uniform microstructure [31].

In the fast regime, the higher source temperature accelerates both vaporization and thermal dissociation of the molten source. This drives TeO_2_ to undergo extensive decomposition, which increases the fraction of elemental Te species in the vapor flux and shifts its composition toward a Te‐enriched state [36]. Simultaneously, the extremely high arrival rate significantly reduces both the inter‐arrival time and the effective surface diffusion length, leading to rapid nucleation across the surface with limited coalescence of individual nuclei [43]. As a result, the adatoms are kinetically quenched upon condensation, forming a microstructure composed of densely nucleated and randomly distributed nanoclusters.

To investigate the microstructural evolution, Raman spectroscopy and grazing‐incidence wide‐angle X‐ray scattering (GIWAXS) were conducted on representative films deposited at 0.1, 1, and 10 Å s^−1^ (Figure 2b,c). In the Raman spectra, three broad peaks were observed near 104, 140, and 175 cm^−1^, corresponding to the *E*_1_, *A*_1_, and *E*_2_ vibrational modes of Te/Se helical chains, respectively. These features are consistent with previous reports in which Se substitution induces a shift toward higher wavenumbers and a broadening of the Raman bands [23, 45]. The blue shift can be attributed to the reduced atomic mass and modified local bonding environment upon Se incorporation, while the peak broadening reflects increased structural disorder and compositional heterogeneity [46, 47].

The 2D GIWAXS images exhibit ring‐shaped diffraction patterns near *q* = 1.62 Å^−1^ (2*θ* = 22.9°) and 1.96 Å^−1^ (2*θ* = 27.8°), corresponding to the (100) and (101) planes, respectively (Figure 2c) [23]. The weak intensities of these rings suggest that the films consist of randomly oriented nanocrystalline domains embedded within a predominantly amorphous matrix, consistent with the cross‐sectional high‐resolution transmission electron microscopy analysis (Figure S8) [48, 49, 50]. Based on the GIWAXS 1D profiles in Figure S9, we estimated the average crystallite sizes using the Scherrer equation [51] to be 6.18, 7.26, and 5.45 nm for the 0.1, 1, and 10 Å s^−1^ films, respectively. Although all films share a near‐amorphous structure, the diffraction intensity differs notably: strongest at 1 Å s^−1^, weaker at 0.1 Å s^−1^, and barely discernible at 10 Å s^−1^. This trend indicates that the extent of nanocrystalline domain formation is maximized at intermediate deposition rates, where surface diffusion and nucleation are balanced. In contrast, it is suppressed at both lower rates due to reduced nucleation density under diffusion‐dominated growth and at higher rates due to kinetically quenched growth.

The surface morphologies of the SeTe–TeO_x_ films were examined by AFM, revealing distinct growth kinetics as a function of deposition rate (insets of Figure 2c and Figure S10a). The 1 × 1 µm^2^ topographic image of the 0.1 Å s^−1^ film shows pronounced protrusions on a compact background. These features may originate from the excessive clustering of Te–Te (or Te–Se) domains, similar to that previously observed in thermally evaporated Te–TeO_x_ films [18]. As the deposition rate increases, these protrusions become less prominent, resulting in a relatively smooth surface at 1 Å s^−^^1^, whereas the film deposited at 10 Å s^−^^1^ exhibits irregularly shaped nanoclusters [30, 31]. Consistently, the calculated root‐mean‐square roughness (*R*_q_) decreases from 5.1 nm at 0.1 Å s^−1^ to a minimum of 1.2 nm at 3 Å s^−1^ and then increases to 2.3 nm at 10 Å s^−1^ (Figure S10b). Overall, the structural and morphological features corroborate the rate‐dependent growth kinetics discussed above. In the following section, the chemical and optoelectronic properties are characterized in detail.

### 2.3 Chemical and Electronic Structure Analysis

**FIGURE 3:**
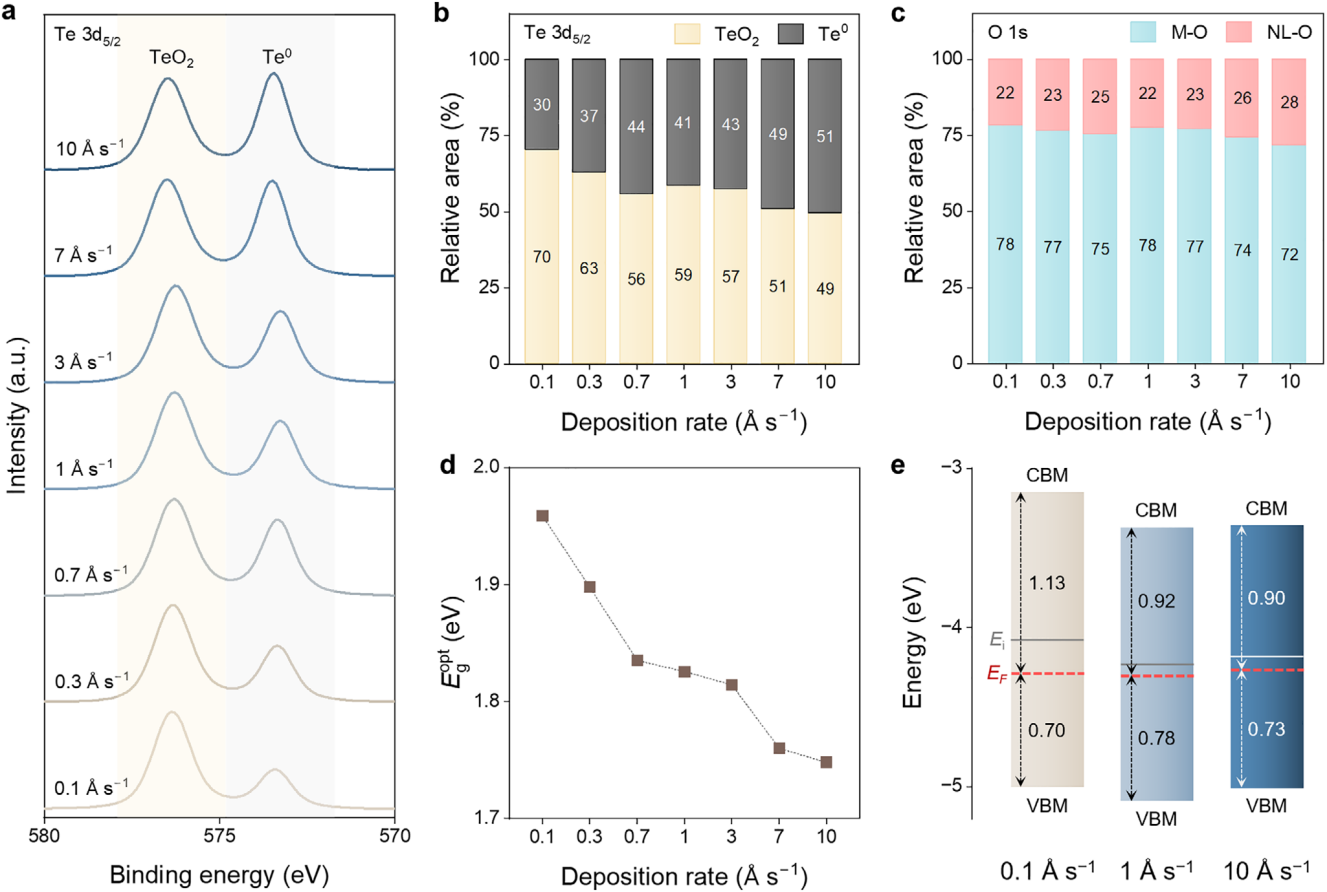
Chemical states and optoelectronic properties. (a) Evolution of Te 3d XPS spectra as a function of deposition rate and (b) corresponding relative area ratios of TeO_2_ and Te^0^ components, normalized to their combined area. (c) Relative area ratios of lattice oxygen (M–O) and non‐lattice oxygen (NL–O) components obtained from O 1s XPS spectra. (d) Optical bandgap ($E_{g}^{\mathrm{opt}}$) extracted from UV–vis spectroscopy. (e) Energy band diagrams for the 0.1, 1, and 10 Å s^−1^ films.

To investigate the chemical states, we acquired X‐ray photoelectron spectroscopy (XPS) spectra of the O 1s, Te 3d, and Se 3d core levels for films deposited at 0.1, 0.3, 0.7, 1, 3, 7, and 10 Å s^−1^, with the detailed deconvolution information presented in Figure S11. Although the highly surface‐sensitive nature of XPS does not fully represent the bulk film, it clearly reveals relative changes in chemical states among the films. In Figure 3a,b, the Te 3d_5/2_ XPS spectra reveal a general increase in the fraction of elemental Te (Te^0^) relative to TeO_2_, indicating that the films contain more metallic Te–Te bonds than oxidized states as the deposition rate increases. The slow regime shows the most pronounced increase in the Te^0^ fraction, rising from 30% to 44%. This trend reflects the strong temperature dependence of reduced Te–O formation under reducing conditions, where the dissociative pathway becomes thermodynamically more favorable relative to the congruent sublimation of TeO_2_. Combined with the low arrival rate, the resulting Te–O‐containing species undergo disproportionation or re‐oxidation during film growth, which yields a higher density of Te–Te bonds or restores Te–O bonding, respectively. As the rate enters the medium regime, the Te^0^ fraction slightly decreases and stabilizes, suggesting a transition toward a more balanced state between the congruent evaporation and the generation of suboxide species_._ However, in the fast regime, the Te^0^ fraction increases again, reaching 51% at 10 Å s^−1^. This behavior is consistent with accelerated decomposition of the source at elevated source temperature, which produces Te‐enriched vapor flux that is kinetically quenched on the deposition surface.

The evolution of Te chemical states is closely followed by the fraction of non‐lattice oxygen (NL–O) observed in the O 1s spectra (Figure 3c). Generally, NL–O species are associated with oxygen‐related structural defects or surface‐adsorbed oxygen species in sub‐stoichiometric oxides [52, 53]. Accordingly, the increase in the NL–O fraction at higher deposition rates suggests the development of a more oxygen‐deficient environment. These observations agree with the qualitative vapor–composition framework proposed in Figure 2a, confirming that the chemical structure is fundamentally governed by the rate‐dependent deposition mechanism. Notably, the atomic composition of Te, Se, and O exhibits only minimal variations across all films (Figure S12). This indicates that our experimental approach enables systematic tuning of the chemical states without causing significant changes in stoichiometry, offering a complementary perspective to previous studies that focused on compositional modification [22, 23].

We performed UV–vis spectroscopy and ultraviolet photoelectron spectroscopy (UPS) to extend the discussion to optoelectronic properties (Figure 3d,e and Figures S13 and S14). The measured $E_{g}^{\mathrm{opt}}$ generally narrows as the Te^0^ fraction increases, because metallic Te–Te states and oxygen deficiency‐induced disorder cause band‐tailing near the absorption onset (Figure 3d) [18, 22, 23, 34]. However, the 0.7 Å s^−1^ film has a wider $E_{g}^{\mathrm{opt}}$ than the 1 Å s^−1^ film despite its higher Te^0^ fraction detected by XPS. We attribute this deviation to possible segregation and aggregation of Te‐rich domains, which are most pronounced at 0.7 Å s^−1^. As discussed earlier, condensed reduced Te–O species are susceptible to disproportionation into elemental Te and TeO_2_, and the relatively long arrival interval may facilitate the formation of localized nanoscopic islands rather than a continuous and homogeneously dispersed network. Such isolated clusters are expected to be ineffective at modulating the bulk optoelectronic properties because they lack sufficient connectivity to form a continuous Te–Te network [54, 55, 56]. This hypothesis is further supported by the anomalous behavior observed in the TFTs fabricated with the 0.7 Å s^−1^ film, which reflects the bulk charge transport characteristics and will be discussed in the next section.

The energy band diagrams further corroborate the electronic structure evolution in the 0.1, 1, and 10 Å s^−1^ films (Figure 3e). Across all the films, the Fermi level (*E*_F_) lies close to the VBM, confirming the intrinsic p‐type characteristics. Furthermore, the VBM appears to be pinned around 5 eV, whereas a pronounced downshift is observed in the CBM. This result is consistent with the previous report on nonlinear bandgap bowing in Te–Se alloys [34], which indicates that increasing the metallic Te content predominantly lowers the CBM rather than elevating the VBM.

### 2.4 Electrical Property Analysis

**FIGURE 4:**
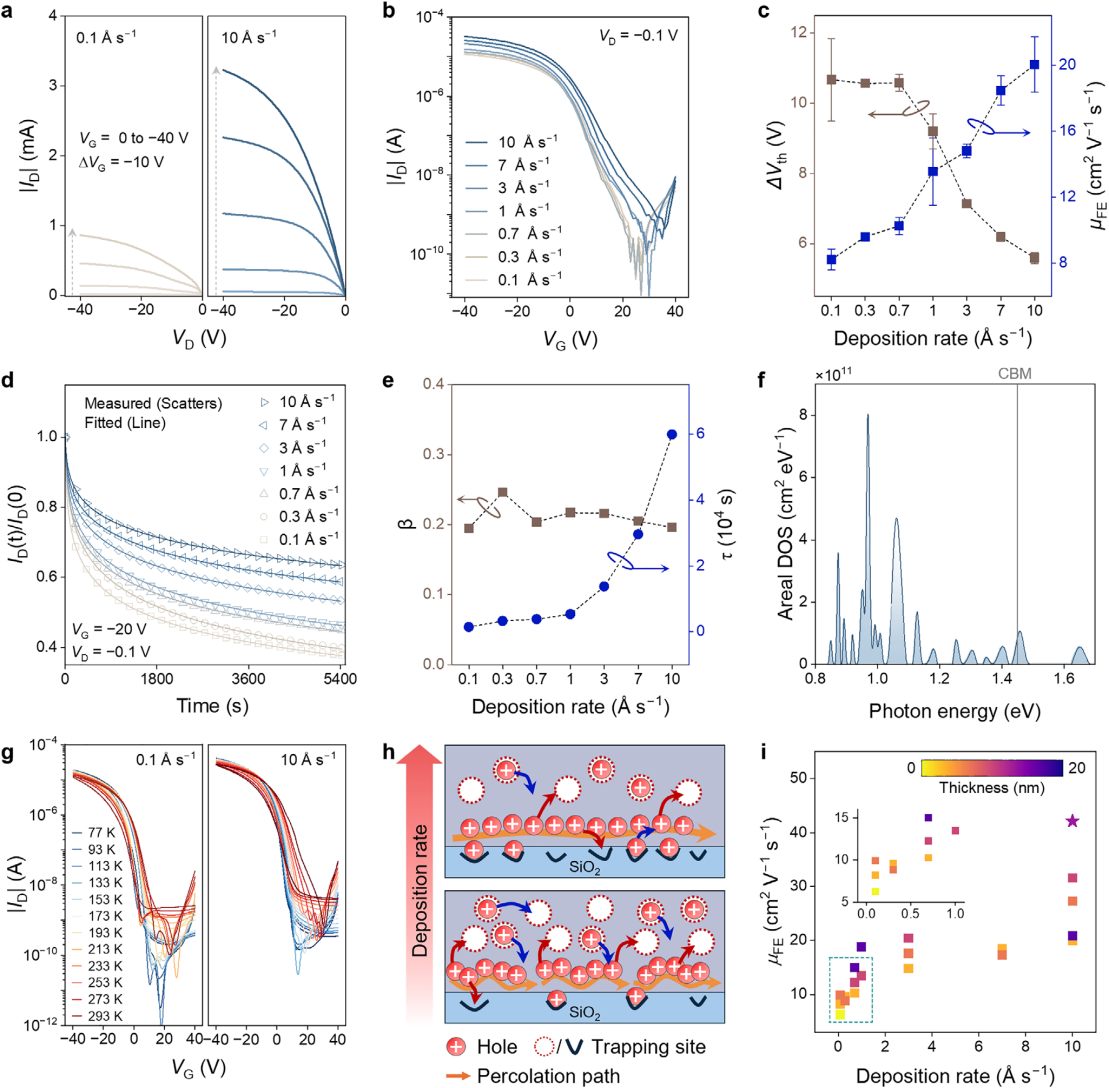
Electrical and charge transport characteristics of SeTe–TeOx TFTs. (a) Output characteristics of 0.1 and 10 Å s^−1^ devices. (b) Transfer characteristics of all seven devices. (c) Linear field‐effect mobility (*µ*_FE_) and hysteresis (*ΔV*_th_); error bars represent the standard deviation of five different channels (Figure S16). (d) Drain current (*I*_D_) decay under negative bias stress for 5400 s. Symbols and solid lines denote measured and stretched exponential fitted data, respectively. (e) Dispersion parameter (*β*) and characteristic time (*τ*) extracted from the fits in (d). (f) Apparent density‐of‐states (DOS) profile obtained from PECCS for the 10 Å s^−1^ device. (g) Temperature‐dependent transfer characteristics of the 0.1 and 10 Å s^−1^ devices measured from 77 to 293 K. (h) Schematic illustration of the charge transport landscape as a function of deposition rate. (i) Thickness‐dependent *µ*_FE_ mapped as a function of deposition rate; the inset shows an enlarged view of the 0.1–1 Å s^−1^ region (dashed box).

We next examine the device characteristics by fabricating bottom‐gate/top‐contact TFTs using films deposited at seven different rates. The output characteristics exhibit typical p‐type behavior and a clear increase in drain current as the deposition rate increases from 0.1 to 10 Å s^−1^ (Figure 4a; full comparison in Figure S15). However, non‐ohmic behavior in the linear region is observed only in the 0.1 Å s^−1^ device, indicating a substantial hole injection barrier. This likely originates from complex factors such as a rougher surface and the formation of an additional oxide layer due to re‐oxidation during the prolonged deposition process, as discussed in Section 2.2, both of which may introduce trap states at a metal/semiconductor interface and hinder efficient hole injection [57].

The transfer characteristics show a monotonic increase in *I*_on_ with the deposition rate (Figure 4b and Figure S16). This overall trend parallels the XPS‐derived increase in the metallic Te fraction, supporting the role of Te–Te‐connected domains as the primary pathways for hole transport [58, 59, 60]. However, the enhancement in *I*_on_ is relatively limited within the slow regime, particularly at 0.7 Å s^−1^. This behavior indicates that the Te^0^ fraction alone does not determine the bulk electronic properties, as discussed in Section 2.3. At 0.7 Å s^−1^, the pronounced segregation of Te‐rich domains is expected to suppress the formation of a dispersive and continuous Te–Te network. The intervening TeO_x_ regions can therefore act as energetic barriers along the charge‐transport path. Consequently, this percolation‐limited charge transport impedes further enhancement in mobility despite the relatively high fraction of metallic states in the film. This interpretation is further supported by the channel sheet resistance (*R*_sh_) extracted using the transfer length method (Figure S17). The *R*_sh_ exhibits a sharp increase from 90.1 to 117.8 kΩ sq^−1^ in the slow regime, followed by a recovery of the expected decreasing trend at higher rates.

Notably, *µ*_FE_ exhibits a strong inverse correlation with *ΔV*_th_ across all devices as summarized in Figure 4c. In dual sweep transfer measurements, *ΔV*_th_ reflects bias‐induced charge trapping and de‐trapping processes occurring within the time window of the gate sweep. Such strong coupling is consistent with a previous low‐frequency noise study, in which carrier‐number fluctuations with correlated mobility fluctuations (CMF) were identified as the dominant noise mechanism [21]. In the CMF framework, trapping and de‐trapping at interface trap states modulate not only the free‐carrier concentration but also the carrier mobility through changes in Coulomb scattering induced by trapped charges [61]. This further suggests that the macroscopic impact of local trapping‐induced perturbations depends on the connectivity of the Te‐derived transport network. Therefore, the observed anticorrelation suggests that the deposition rate‐dependent charge transport characteristics are closely coupled to variations in the underlying trapping dynamics.

Charge trapping over extended timescales was evaluated by applying negative bias stress for 5,400 s while monitoring the resulting *I*_D_ decay (Figure 4d). The progressive suppression of *I*_D_ decay with increasing deposition rate demonstrates that the bias‐induced trapping exerts a strong influence on the percolation‐limited transport network formed at slow deposition rates, whereas its impact is substantially reduced in a well‐percolated network formed at high rates. The *I*_D_ decay curves were fitted using a stretched exponential function to extract the dispersion parameter (*β*) and the characteristic time (*τ*) (Figure 4e) [62, 63]. The similar *β* values for all devices imply a comparable degree of dispersive trapping kinetics across the devices. In contrast, *τ* increases gradually in the slow regime and rises more rapidly in the medium and fast regimes, indicating that mobile carriers remain conductive for longer periods before being affected by trapping at higher rates. Together with the reduced decay magnitude, this result supports more persistent channel conduction and a diminished effective influence of bias‐induced trapping in the well‐percolated devices.

To further examine the charge trapping kinetics, we tracked the transfer characteristics evolution of the 0.1, 1, and 10 Å s^−1^ devices during prolonged bias stress and their recovery after stress removal (Figure S18). Under bias stress, the 10 Å s^−1^ device exhibits minimal changes in transfer characteristics, including *I*_on_, *V*_th_, and *SS*, whereas the 0.1 and 1 Å s^−1^ devices undergo pronounced *I*_on_ decreases, negative *V*_th_ shifts, and *SS* degradation. This contrasting behavior highlights the prominent role of the cumulative occupation of kinetically slow, bias‐active trap states in the slow rate devices. Accordingly, the normalized *V*_th_ relaxation summarized in Figure S19 reveals slower recovery in the 0.1 and 1 Å s^−1^ devices than in the 10 Å s^−1^ device. In addition, the 0.1 and 1 Å s^−1^ devices show a strong dependence on the scanning step, in which smaller steps (or longer measurement durations) degrade transfer characteristics (Figure S20). Collectively, these bias‐related measurements indicate enhanced robustness against kinetically slow trapping at higher deposition rates.

As a complementary spectroscopic measurement, photo‐excited charge collection spectroscopy (PECCS) was employed to probe the trap states near the semiconductor/dielectric interface [64]. During the measurement, the channel was sequentially illuminated with monochromatic light over a broad wavelength range of 1500–300 nm to photo‐excite carriers trapped at the corresponding energies. The resulting photo‐induced *V*_th_ shifts were then used to extract the apparent areal density of states (DOS) profile as a function of photon energy. A small drain voltage of −0.1 V was applied to suppress bulk‐transport contributions and maintain an interface‐sensitive response. As shown in Figure 4f, the 10 Å s^−1^ device exhibits a high apparent DOS over a broad energy range, indicating a substantial population of optically addressable interfacial states, whereas the 0.1 and 1 Å s^−1^ devices show comparatively lower DOS values (Figure S21). Despite their lower apparent interfacial DOS, the 0.1 and 1 Å s^−1^ devices exhibit stronger bias instability, suggesting a significant contribution from kinetically slow traps associated with the channel bulk or percolation‐limited transport pathways. Furthermore, common DOS features are observed between 0.83 and 1.2 eV, which may reflect oxygen‐related defects and interstitial Te/Se arising from structural or chemical variations in the films [19, 20]. Complementary measurements and computational analyses would enable a more rigorous discussion of the origins of these subgap features.

Beyond the extracted DOS profiles, the repeated transfer sweeps involved in PECCS provide additional insight into bias‐induced trapping dynamics, as evidenced by the initial reverse *V*_th_ shift at photon energies below ∼2.1 eV (Figure S22). This indicates that bias‐induced re‐trapping dominates over photo‐induced de‐trapping in this subgap region. Its stronger manifestation at lower deposition rates further supports the higher sensitivity of percolation‐limited devices to bias‐induced trapping.

Temperature‐dependent measurements of the 0.1 and 10 Å s^−1^ devices further elucidate the deposition‐rate‐dependent trapping landscape (Figure 4g). Both devices exhibit comparable decreases in *µ*_FE_ as the temperature increases from 77 to 293 K (Figure S23a), indicating that band‐like transport is maintained in both devices despite their different degrees of transport path connectivity [60]. In contrast, the significant temperature dependence of *SS* and *ΔV*_th_ was observed in the 10 Å s^−1^ device (Figure S23b–c). Specifically, its *SS* and *ΔV*_th_ increase by approximately 5.0 and 5.1‐fold, respectively, compared with 3.6 and 2.5‐fold increases in the 0.1 Å s^−1^ device. In AOSs, localized states near the band edge or within the band tail can participate in thermally activated carrier trapping and release, thereby broadening the subthreshold transition and increasing hysteresis [21]. Therefore, these results suggest a greater population of thermally accessible near‐band‐edge states in the 10 Å s^−1^ device than in the 0.1 Å s^−1^ device. We attribute these states to increased local structural and chemical disorder at high deposition rates, including Te enrichment and oxygen‐deficiency‐induced band‐tail formation.

Integrating the chemical, microstructural, and electrical analyses, Figure 4h illustrates the deposition‐rate‐dependent charge‐transport landscape. Within a common band‐like transport regime, low deposition rates produce a fragmented Te–Te network because the Te–O‐containing vapor flux limits the formation of continuous percolative pathways. The resulting percolation‐limited channel is highly sensitive to kinetically slow traps associated with the channel bulk and critical transport bottlenecks. In contrast, the increased elemental Te fraction and shorter adatom burial time at high rates form a well‐connected network that mitigates the macroscopic influence of slow trapping and sustains stable charge transport. This reduced sensitivity to slow trapping nevertheless coexists with a larger population of thermally responsive interfacial and near‐band‐edge states, potentially arising from increased interfacial and structural disorder [34, 35].

To fully exploit the potential of the SeTe–TeO_x_ system, we introduce film thickness as an additional parameter. By systematically tuning both thickness and deposition rate, we identify a synergetic effect between the two parameters, that is, increasing the film thickness at faster deposition rates yields a higher *µ*_FE_, reaching a maximum value of 42.1 cm^2^ V^−1^ s^−1^ for a 19 nm film deposited at 10 Å s^−1^ (Figure 4i and Figure S24). These conditions effectively suppress *Δ* *V*_th_, but this improvement is accompanied by increases in *I*_off_ and *SS*, indicating a trade‐off between bias stability and switching performance. Increasing the film thickness can reduce the relative influence of surface and interface disorder while enhancing lateral and vertical connectivity among Te‐rich transport domains. The resulting mobility enhancement therefore likely arises from the combined effects of thickness‐enhanced connectivity and deposition‐rate‐induced formation of an effective Te‐rich transport network, rather than from simple geometric thickness scaling. However, excessive deposition rates or thicknesses deteriorate both *µ*_FE_ and the switching characteristics and ultimately lead to metal‐like behavior. This degradation likely arises from the excessive Te–Te metallic states and inherent structural instability, which induce a large density of defect states distributed over a broad energy range.

## 3 Conclusion

We demonstrated that the thermal deposition rate is a simple yet powerful process parameter for tuning the properties of SeTe–TeO_x_ films. The combined thermodynamic and ex situ analyses support a rate‐dependent noncongruent vaporization framework for the blended Se/TeO_2_ source, in which the contribution of elemental Te species increases with deposition rate. The resulting changes in chemical states and microstructure modulate the connectivity of Te‐derived transport pathways and, consequently, the charge‐transport characteristics, trapping kinetics, and bias stability of the thin‐film transistors. More broadly, the proposed mechanism suggests that tailored power ramps, shutter timing, and multistage deposition profiles could be used to mitigate or deliberately exploit the intrinsic vertical compositional gradient as an additional degree of freedom in material design. However, fully suppressing volatility‐driven compositional inhomogeneity will likely require further development of deposition approaches that independently control the constituent fluxes or employ fundamentally different film‐forming mechanisms. We anticipate that this study will broaden the understanding of this emerging semimetal‐in‐oxide material system and facilitate the development of reliable, high‐performance p‐type amorphous oxide semiconductors for all‐oxide electronics.

## 4 Experimental Section

### 4.1 Deposition of SeTe–TeOx Films at Controlled Rates

A mixture of 400 mg TeO_2_ (>98%, Tokyo Chemical Industry) and 12 mg Se (>99.9%, Tokyo Chemical Industry) was loaded into a tungsten boat. The deposition rate and film thickness were monitored with a quartz‐crystal microbalance (QCM). The boat–substrate distance was around 30 cm, and all depositions were carried out under 6 × 10^−6^ Torr. The power was increased at 0.1 W min^−1^ until the QCM began to read rate and was then ramped linearly to reach one of seven target deposition rates (0.1, 0.3, 0.7, 1, 3, 7, and 10 Å s^−1^). The shutter was closed when the film thickness reached 15 nm.

### 4.2 Film Characterization

GIWAXS images were acquired at the 3C beamline of the Pohang Accelerator Laboratory (PAL), Korea. Surface morphology was characterized using an NX‐10 atomic force microscope (Park Systems) in non‐contact mode. XPS and UPS were conducted on a NEXSA G2 system (Thermo Fisher Scientific) with an Al Kα X‐ray source (1486.6 eV) and a He I discharge lamp (21.2 eV). To quantify atomic composition, the areas of Te 3d and Se 3d were normalized using relative sensitivity factors of 30.58 and 2.294, respectively. UV–vis–NIR transmission spectra from λ = 330 to 2000 nm were collected with a JASCO V‐770 spectrophotometer. Cross‐sectional high‐resolution transmission electron microscopy images were acquired using a JEM‐2100F microscope (JEOL).

### 4.3 Fabrication of TFTs

TFTs were fabricated on SiO_2_/Si substrates. A heavily doped p‐type Si wafer served as the gate electrode, and 100 nm thermally grown SiO_2_ served as the gate dielectric. Substrates were cleaned by sequential ultrasonication in acetone, isopropanol, and deionized water for 15 min each, dried under N_2_ flow, then annealed at 80°C. Surface treatment was performed using a UV–ozone cleaner for 10 min. SeTe–TeO_x_ films were deposited as described above, then annealed on a hot plate at 225°C for 30 min in air. Ni source and drain electrodes (40 nm) were thermally evaporated at ∼1 Å s^−^^1^ and patterned with a shadow mask. The device geometry was L = 80 µm and W = 1000 µm.

### 4.4 Electrical Characterization

Electrical properties of the TFT devices were evaluated under dark and vacuum conditions (∼1 × 10^−^^3^ Torr) at room temperature using a Keithley 4200 semiconductor parameter analyzer. The field‐effect mobility (*µ*_FE_) in the linear regime was extracted from $\mu_{\mathrm{FE}}=\frac{L}{W\;C_{i}\;V_{D}}\;\frac{dI_{D}}{dV_{G}}$, where *L* and *W* are the channel length and width, and *C*_i_ is the gate dielectric capacitance per unit area. The subthreshold swing (*SS*) was calculated as $SS=\frac{dV_{G}}{d(\log_{10}\left|I_{D}\right|)}$ . Hysteresis (*Δ* *V*_th_) was defined as the threshold‐voltage difference between forward and backward sweeps ( *Δ* *V*_th_ = *V*_th.forward_  − *V*_th.backward_). For transfer length method (TLM) analysis, devices with *L* = 30, 80, 130, and 180 µm with *W* = 1000 µm were used. The sheet resistance *R*_sh_ was obtained from the slope of *R*_tot_ versus *L* using $R_{\mathrm{tot}}=\frac{R_{sh}}{W}\;L+2R_{c}$.

### 4.5 Photo‐Excited Charge Collection Spectroscopy (PECCS)

PECCS measurements were conducted under the same conditions as the other electrical characterizations. Monochromatic light, generated using a 500 W Hg (Xe) arc lamp coupled to a grating monochromator, was incident on the devices for 8 s at 10 nm intervals from 1500 to 300 nm. After each illumination step, transfer curves were recorded in the dark, and the threshold voltage (*V*_th_) was extracted using the constant‐current method. The energy‐resolved density of states was calculated from $D_{\mathrm{it}}\;\left(E_{v}+\varepsilon \right)=-\frac{C_{i}}{q}\frac{\partial V_{\mathrm{th}}\left(\varepsilon \right)}{\partial \varepsilon }$, where *ε* is the photon energy and *q* is the elementary charge.

### 4.6 Other Characterization

TGA and DSC were performed with an SDT 650 simultaneous TGA/DSC analyzer (TA Instruments) under N_2_ at a heating rate of 10 °C min^−^^1^ from 30 to 1300 °C.

## Author Contributions

**Seongmo Kang**: conceptualization, investigation, methodology, validation, writing – original draft, writing – review and editing, formal analysis, data curation, visualization. **Jeong‐Min Seo**: conceptualization, visualization, writing – original draft. **Hyungyu Choi**: methodology, investigation, formal analysis. **Yunseo Song**: methodology, data curation, investigation. **Tae Woong Yoon**: investigation, methodology. **Tae Woong Han**: methodology, investigation. **Hoseong Shin**: validation, conceptualization. **Jiyun Lee**: conceptualization, visualization. **Boseok Kang**: conceptualization, visualization, funding acquisition, supervision, project administration.

## Conflicts of Interest

The authors declare no conflicts of interest.

## Supporting information




**Supporting File**: advs77618‐sup‐0001‐SuppMat.docx.

## Data Availability

The data that support the findings of this study are available on request from the corresponding author. The data are not publicly available due to privacy or ethical restrictions.
