# Supplementary material for: Deposition Rate‐Controlled Functional Evolution of p‐Type Selenium‐Alloyed Tellurium–Tellurium Oxide Films for Thin‐Film Transistors
Source: Adv Sci (Weinh). 2026 Sep 8:e77618. Online ahead of print. doi: 10.1002/advs.77618 (PMC13554495; doi:10.1002/advs.77618)
Supplement: Supplementary file 1 [file ADVS-9999-e77618-s001.docx]

**Deposition Rate-Controlled Functional Evolution of p-Type Selenium-Alloyed Tellurium–Tellurium Oxide Films for Thin-Film Transistors**

*Seongmo Kang, Jeong-Min Seo, Hyungyu Choi, Yunseo Song, Tae Woong Yoon, Tae Woong Han, Hoseong Shin, Jiyun Lee, and Boseok Kang**

S. Kang, JM. Seo, H. Choi, Y. Song, TW. Yoon, TW. Han, H. Shin, J. Lee, Prof. B. Kang

SKKU Advanced Institute of Nanotechnology (SAINT) and Department of Nano Science and Technology, Sungkyunkwan University, 2066 Seobu-ro, Jangan-gu, Suwon, Gyeonggi-do, 16419, Korea

***Email: bskang88@skku.edu

Prof. B. Kang

Department of Nano Engineering and Department of Semiconductor Convergence Engineering, Sungkyunkwan University, 2066 Seobu-ro, Jangan-gu, Suwon, Gyeonggi-do, 16419, Korea

Keywords: p-type oxide semiconductors; tellurium oxide; thermal evaporation; deposition rate; thin-film transistors

**1. Supporting Notes**

**Note S1. Qualitative relationship between electrical power and deposition rate.**

Although the basic mechanism of thermal evaporation is conceptually intuitive, film growth is governed by several interdependent variables that dictate crystallinity, stoichiometry, and surface morphology. These variables include the deposition rate, the chamber pressure, the substrate temperature, and the system configuration (e.g., source-to-substrate distance and the incident angle of the adatoms). Among these, the deposition rate is the most critical variable because it reflects the combined kinetic and thermodynamic behavior of the source material and the resulting vapor flux. Therefore, we systematically vary the deposition rate while keeping the other parameters fixed, and we investigate the evolution of the Se-alloyed Te–TeO_x_ film’s chemical, microstructural, and electrical properties.

In this note, we describe the relationship between the applied electrical power and the deposition rate in three steps using well-established physical relationships. The purpose of this note is not to derive a rigorous quantitative relationship between electrical power and deposition rate, but rather to provide a qualitative physical framework connecting the supplied power, source temperature, vapor pressure, and resulting deposition flux.

**Step 1. Power and Temperature Relation**: The electrical power (energy per unit time) generated by the resistance of the boat is defined by Joule’s heating law ($P=I\times V)$. For the qualitative framework considered here, we restrict the thermal system to the resistively heated boat. Under high-vacuum conditions, convective heat loss is negligible, and at sufficiently high temperatures, radiative heat loss from the boat is assumed to dominate the overall heat dissipation. Accordingly, the steady-state power balance of the boat can be approximated by the Stefan–Boltzmann relation:

$\boldsymbol{P=\varepsilon\sigma A(}\boldsymbol{T}_{\mathbf{boat}}^{\boldsymbol{4}}\boldsymbol{-}\boldsymbol{T}_{\mathbf{amb}}^{\boldsymbol{4}}\boldsymbol{)}$,

(1)

where *ε* is the emissivity of the boat metal, *σ* is the Stefan-Boltzmann constant, *A* is the surface area of the boat, *T*_boat_ is the boat temperature, and *T*_amb_ is the ambient temperature. At *T*_boat_ ≫ *T*_amb_, this relation gives the approximate scaling

$\boldsymbol{T}_{\mathbf{boat}}\boldsymbol{\propto}\boldsymbol{P}^{\boldsymbol{1/4}}$.

(2)

Because the source material is in direct thermal contact with the boat, its temperature is expected to increase correspondingly with *T*_boat_, although an exact equality between the boat and source temperatures is not assumed.

**Step 2. Temperature and Vapor Pressure Relation**: According to the Clausius–Clapeyron equation, the temperature dependence of vapor pressure is expressed as:

$\frac{\boldsymbol{dp}}{\boldsymbol{dT}}\boldsymbol{=}\frac{\boldsymbol{\Delta H}}{\boldsymbol{T\Delta V}}$,

(3)

where *ΔH* is the enthalpy of vaporization and *ΔV* is the volume change. For an ideal vapor, we can approximate *ΔV* ≈ *V*_gas_ ≈ *RT*/*p*, which leads to

$\frac{\boldsymbol{dln}\left( \boldsymbol{p} \right)}{\boldsymbol{dT}}\boldsymbol{=}\frac{\boldsymbol{\Delta H}}{\boldsymbol{R}}\frac{\boldsymbol{1}}{\boldsymbol{T}^{\boldsymbol{2}}}$.

(4)

Assuming *ΔH* is approximately constant over the relevant temperature range, integration gives

$\ln\left( \boldsymbol{p} \right)\boldsymbol{=-}\frac{\boldsymbol{\Delta H}}{\boldsymbol{RT}}\boldsymbol{+C}$,

(5)

where *R* is the gas constant, and *C* is an integration constant. Because the source material is in direct thermal contact with the boat, we qualitatively assume that the source temperature follows the boat temperature, i.e., *T*_source_ ∝ *T*_boat_ ∝ *P*^1/4^. Substituting **Equation (2)**, the relationship can be simplified as:

$\ln\left( \boldsymbol{p} \right)\boldsymbol{\approx-A'}\boldsymbol{P}^{\boldsymbol{-1/4}}\boldsymbol{+}\boldsymbol{C}^{\boldsymbol{'}}$,

(6)

or equivalently,

$\boldsymbol{p\approx}\boldsymbol{p}^{\boldsymbol{*}}\mathbf{exp}\boldsymbol{(-A'}\boldsymbol{P}^{\boldsymbol{-1/4}}\boldsymbol{)}$,

(7)

where *A′* and *p*^*^ are constants incorporating *ΔH*/*R*, the proportionality between source temperature and supplied power, and the integration constant.

**Step 3. Deposition Rate and Geometry***:* The Hertz–Knudsen equation relates the evaporation rate (*J*), which is the number of particles leaving the surface per unit area per unit time, to the vapor pressure as:

$\boldsymbol{J=}\frac{\boldsymbol{\alpha}\left( \boldsymbol{p-}\boldsymbol{p}_{\mathbf{amb}} \right)}{\sqrt{\boldsymbol{2}\boldsymbol{\pi}\boldsymbol{m}_{\mathbf{0}}\boldsymbol{k}_{\mathbf{B}}\boldsymbol{T}_{\mathbf{source}}}}$,

(8)

where *α* is the evaporation coefficient, *p* is the vapor pressure, *p*_amb_ is the ambient partial pressure, *m*_0_ is the molecular mass, and *k*_B_ is the Boltzmann constant. Under high vacuum, *p* ≫ *p*_amb_, and If we assume nearly ideal evaporation (every molecule on the surface evaporates and does not return to the source, *α* ≈ 1), this simplifies to

$\boldsymbol{J\approx}\frac{\boldsymbol{p}}{\sqrt{\boldsymbol{2}\boldsymbol{\pi}\boldsymbol{m}_{\mathbf{0}}\boldsymbol{k}_{\mathbf{B}}\boldsymbol{T}_{\mathbf{source}}}}$*.*

(9)

Considering the geometric factor where the flux reaching the substrate follows the inverse square law (1/*r*^2^), the deposition rate (*R*_dep_) is expressed as:

$\boldsymbol{R}_{\mathbf{dep}}\boldsymbol{\propto}\frac{\boldsymbol{J}}{\boldsymbol{r}^{\boldsymbol{2}}}\boldsymbol{\propto}\frac{\boldsymbol{p}}{\boldsymbol{r}^{\boldsymbol{2}}\sqrt{\boldsymbol{T}_{\mathbf{source}}}}$.

(10)

Although *J* is inversely proportional to $\sqrt{T_{\mathrm{source}}}$, the exponential dependence of vapor pressure is far more dominant. For a qualitative discussion, we therefore treat $1/\sqrt{T_{\mathrm{source}}}$ as an approximately constant prefactor and write

$\boldsymbol{R}_{\mathbf{dep}}\boldsymbol{\approx}\frac{\boldsymbol{R}_{\boldsymbol{0}}}{\boldsymbol{r}^{\boldsymbol{2}}}\boldsymbol{p}$,

(11)

where *R*_0_ is a geometry- and material-dependent constant. Combining this with **Equation (7)**, the following approximate relationship is obtained:

$\boldsymbol{R}_{\mathbf{dep}}\boldsymbol{(P)\approx}\frac{\boldsymbol{R}_{\boldsymbol{0}}\boldsymbol{p}^{\boldsymbol{*}}}{\boldsymbol{r}^{\boldsymbol{2}}}\mathbf{exp}\boldsymbol{(-}\boldsymbol{A}^{\boldsymbol{'}}\boldsymbol{P}^{\boldsymbol{-1/4}}\boldsymbol{)}$.

(12)

**Note S2. Studies on thermodynamic behavior of TeO_2_ under actual deposition conditions.**

TeO_2_ is well-known for its noncongruent evaporation characteristics. At elevated temperatures, it undergoes a series of complex reactions, including phase transformation, polymerization, and partial decomposition. Because of the significant utility of TeO_2_ in nuclear technology, its thermochemical behaviors have been extensively studied and thoroughly reviewed by several authors.^[1-5]^ For a simple and practical discussion, we specifically adopted the vaporization study on TeO_2_ reported by Lakshmi Narasimhan et al.^[4]^

In this note, we integrate our experimental findings with a thermodynamic analysis to elucidate the vaporization pathways of heated TeO_2_, focusing on the major reduced Te-containing species (TeO and Te (Te_2_)). Our interest is in the competition between congruent sublimation and reduction to suboxide species during the vacuum deposition process.

**Figure N1.** Pressure-derived Gibbs terms calculated from the equilibrium partial pressures in **Equations (1–3)** of Ref. [4], together with the O_2_-pressure-dependent contribution (green line) for TeO formation.

Lakshmi Narasimhan et al. determined the vapor pressure (*p*) and temperature (*T*) relationships for TeO_2_(g), TeO(g), and Te_2_(g) in the range of 805–905 K using Knudsen effusion mass spectrometry.^[4]^

|  | $\mathbf{log}_{\boldsymbol{10}} \boldsymbol{p(}\mathbf{Te}\mathbf{O}_{\mathbf{2}}\boldsymbol{, g)}\left[ \mathbf{Pa} \right]\boldsymbol{=(15.021\pm0.085)-}\frac{\boldsymbol{13988\pm72}}{\boldsymbol{T}\left[ \mathbf{K} \right]}$, | (1) |
| --- | --- | --- |
| \| $\mathbf{log}_{\boldsymbol{10}} \boldsymbol{p(}\mathbf{Te}\mathbf{O}_{\mathbf{2}}\boldsymbol{, g)}\left[ \mathbf{Pa} \right]\boldsymbol{=(15.021\pm0.085)-}\frac{\boldsymbol{13988\pm72}}{\boldsymbol{T}\left[ \mathbf{K} \right]}$, \| (1) | $\mathbf{log}_{\boldsymbol{10}} \boldsymbol{p(}\mathbf{TeO}\boldsymbol{, g)}\left[ \mathbf{Pa} \right]\boldsymbol{=(14.535\pm0.093)-}\frac{\boldsymbol{13738\pm79}}{\boldsymbol{T}\left[ \mathbf{K} \right]}$, | (2) |
|  | $\mathbf{log}_{\boldsymbol{10}} \boldsymbol{p(}\mathbf{Te}_{\mathbf{2}}\boldsymbol{, g)}\left[ \mathbf{Pa} \right]\boldsymbol{=}\left( \boldsymbol{13.444\pm0.178} \right)\boldsymbol{-}\frac{\boldsymbol{13443\pm151}}{\boldsymbol{T}\left[ \mathbf{K} \right]}$. | (3) |

At 900 K, these equations yield equilibrium pressures of ~0.3, ~0.19, and 0.03 Pa, respectively. These values indicate that reduced Te-containing species can have partial pressures comparable to that of TeO_2_(g). For comparative visualization, the corresponding pressure-derived Gibbs terms, $-RT\ln\left( p_{O_{2}}/p^{\circ} \right)$, are plotted in **Figure N1**. For the congruent sublimation reaction, TeO_2_(s) → TeO_2_(g), this term directly corresponds to the standard Gibbs free energy of sublimation:

$\boldsymbol{\Delta G}_{\mathbf{sub}}^{\boldsymbol{\circ}}\boldsymbol{=-RT}\ln\left( \frac{\boldsymbol{p}_{\mathbf{eq}}}{\boldsymbol{p}^{\boldsymbol{\circ}}} \right)$,

(4)

where *R* is the gas constant (~8.314 J mol^−1^ K^−1^), *p*_eq_ and *p*∘ are the equilibrium and standard pressure, respectively. Substituting the calculated equilibrium pressure, ${\Delta G}_{\mathrm{sub}}^{\circ}$ is ~95 kJ mol^−1^ at 900 K, consistent with the low equilibrium vapor pressure of molecular TeO_2_. This relatively large positive value is also qualitatively consistent with the high enthalpy of sublimation measured by transpiration (~255 kJ mol^−1^ at 936 K)^[3]^ and with the negligible weight loss observed before the melting point in our TGA data (**Figure 1e**).

However, reductive pathways can be facilitated under high vacuum, open-system conditions, where volatile species, including oxygen-containing products, are continuously removed. For example, for the primary dissociation reaction, TeO_2_(s) → TeO(g) + ½O_2_(g), the Gibbs free energy at nonstandard partial pressures can be expressed as:

$\boldsymbol{\Delta}\boldsymbol{G}_{\mathbf{diss}}\left( \boldsymbol{T,}\boldsymbol{p}_{\mathbf{O}_{\mathbf{2}}} \right)\boldsymbol{=\Delta}\boldsymbol{G}_{\mathbf{diss}}^{\boldsymbol{\circ}}\left( \boldsymbol{T} \right)\boldsymbol{+RT}\ln\left[ \left( \frac{\boldsymbol{p}_{\mathbf{TeO}}}{\boldsymbol{p}^{\boldsymbol{\circ}}} \right)\left( \frac{\boldsymbol{p}_{\mathbf{O}_{\mathbf{2}}}}{\boldsymbol{p}^{\boldsymbol{\circ}}} \right)^{\boldsymbol{1/2}} \right]$.

(5)

To illustrate the specific effect of the oxygen-deficient deposition environment, we consider the $p_{O_{2}}$-dependent contribution, $1/2RT\ln\left( p_{O_{2}}/p^{\circ} \right).$ Taking the total chamber pressure (6 ×10^−6^ Torr) as an upper-bound estimate of $p_{O_{2}}$, this term reaches approximately −70 kJ mol^−1^ at 900 K (**Figure N1**). This substantial negative contribution thermodynamically favors the dissociation pathway toward reduced Te-containing species under oxygen-deficient conditions. In addition, direct contact between the TeO_2_ powder and the highly refractory tungsten boat may further facilitate interfacial reduction or decomposition.

Based on this thermodynamic analysis, we can interpret the pronounced increase in the relative Te^0^ fraction observed within the slow regime (0.1, 0.3, and 0.7 Å s^−1^) shown in **Figure 3b**. As the source temperature increases, the magnitude of the negative $p_{O_{2}}$-dependent contribution to the dissociation reaction increases, thereby progressively favoring the formation of reduced Te-containing species under oxygen-deficient conditions. Accordingly, the lowest-rate condition (0.1 Å s^−1^), corresponding to the lowest source temperature within this regime, experiences the weakest thermodynamic bias toward dissociation and therefore retains a relatively greater contribution from molecular TeO_2_ vaporization.

As the source temperature increases to the melting point and the system enters the medium regime (1 and 3 Å s^−1^), vaporization of molecular TeO_2_ becomes kinetically more accessible from the liquid phase than from the solid. This recovery of congruent evaporation is attributed to two plausible mechanisms: (1) the fluidity of the liquid phase rapidly suppresses the formation of a reduced surface layer, and (2) oxygen release increases the local oxygen potential near the melt surface. Together, these factors partially restore congruent evaporation while decomposition into suboxides remains active, which is consistent with the slight decrease of Te^0^ fraction observed in the XPS analysis (Figure 3b).

In the fast regime (7 and 10 Å s^−1^), the substantial thermal energy input promotes both vaporization and thermal dissociation of the molten source. The vapor pressure of Te-containing species increase strongly with temperature, while dissociative pathways become increasingly accessible under the oxygen-deficient, open-system conditions. This enhanced thermal dissociation can generate elemental Te-containing species (e.g., TeO_2_ → Te(g) + O_2_(g)), shifting the vapor composition towards an elemental Te-enriched state.

**2. Supporting figures**

**Figure S1.** (a) Atomic force microscopy (AFM) images and corresponding line profiles of films deposited at rates from 0.1 to 10 Å s⁻¹. (b) Quartz-crystal microbalance (QCM) tooling factors calibrated for each deposition rate to maintain comparable film thicknesses.

**Figure S2.** (a) Photograph of the film deposited at a rate of 10 Å s⁻¹ on a 5 × 5 cm² substrate. (b) The substrate was divided into twenty-five 1 × 1 cm² regions for AFM thickness measurements. (c) Spatial thickness-distribution map reconstructed from the 25-point measurements.

**Figure S3.** Original photograph of Figure 1c, showing films deposited on a glass substrate without Se (left) and with Se (right).

**Figure S4.** (a) Thermogravimetric analysis (TGA) and differential scanning calorimetry (DSC) curves of the Se (12 mg) and TeO_2_ (400 mg) blended source. (b) TGA curve of the post-evaporation remnant; the inset shows an enlarged view of 200–500 ℃ region (dashed box).

**Figure S5.** Normalized intensity comparison of Te 3d and Se 3d X-ray photoelectron spectroscopy (XPS) spectra of (a) 3 nm, (b) 5 nm, (c) 10 nm, and (d) 15 nm Se-alloyed Te–TeO_x_ films. The relative sensitivity factors were used for quantifying atomic composition, which are 30.58 for Te 3d and 2.294 for Se 3d, respectively.

**Figure S6.** (a) Cross-sectional scanning transmission electron microscopy (STEM) image and (b) corresponding energy-dispersive X-ray spectroscopy (EDS) elemental maps of silicon, selenium, and tellurium. The white box indicates the analyzed region.

**Figure S7.** Original photographs of **Figure 1g**. (a) pre- and (b) post-evaporation boats.

**Figure S8.** Cross-sectional high-resolution transmission electron microscopy (HR-TEM) images of films deposited at (a, b) 0.1 Å s⁻¹ and (c, d) 10 Å s⁻¹. The orange dashed lines indicate the film boundaries. The red and white dashed boxes indicate the largely crystalline and amorphous domains, respectively. Panels (a,c) show low-magnification views, while (b,d) present enlarged regions exhibiting predominantly amorphous features on the left and more pronounced crystalline features on the right.

**Figure S9.** Grazing-incidence wide-angle X-ray scattering (GIWAXS) one-dimensional profiles for the representative films deposited at 0.1, 1, and 10 Å s^−1^.

**Figure S10.** (a) Atomic force microscopy (AFM) images and (b) root-mean-square roughness (*R*_q_) summary plot of the seven films.

**Figure S11.** Deconvoluted XPS spectra for the seven films. (a) O 1s spectrum consisting of metal–oxygen (M–O) and non-lattice oxygen (NL–O) components. (b) Te 3d spectrum consisting of Te^6+^, Te^4+^, TeO_x_ (suboxide), Te^0^ components. (c) Se 3d spectrum consisting of Se 3d_3/2_ and Se 3d_5/2_ components.

**Figure S12.** Summary plot of the atomic composition for the seven films. The areas of Te 3d, Se 3d, and O 1s core-level spectra are normalized using relative sensitivity factors of 30.58, 2.294, and 2.93, respectively.

**Figure S13.** (a) UV–vis transmittance spectra and (b) corresponding Tauc plots of the seven films. (c) Enlarged view of the cyan-boxed region in (b), showing the selected linear regions and extrapolated fitting lines used to determine the apparent optical bandgaps for comparative evaluation among the films.

**Figure S14.** Ultraviolet photoelectron spectroscopy (UPS) analysis of the three representative films. The intersections of the dashed lines indicate (a) *E*_f_ – *E*_VBM_ and (b) the secondary cutoff, respectively.

**Figure S15.** Output characteristics of the devices fabricated with the seven films. The gate voltage (*V*_G_) decreased from 0 V to −40 V at an increment of −10 V.

**Figure S16.** Transfer characteristics of the five channels for each of the seven devices. The curves were recorded in the linear regime (*V*_D_ = −0.1 V).

**Figure S17.** (a) Normalized total resistance as a function of channel length measured using the transfer length method (TLM) and (b) calculated sheet resistance (*R*_sh_) at *V*_G_ = −40 V of the seven devices.

**Figure S18.** Recovery test results for the three representative devices. (a) Evolution of transfer characteristics under negative bias (*V*_D_ = −0.1 V and *V*_G_ = −20 V) for 1,890 s. (b) Recovery behavior for 3,810 s after the bias stress.

**Figure S19.** Normalized threshold voltage (*V*_th_) shift behavior during bias stress and subsequent recovery.

**Figure S20.** Evolution of the transfer characteristics of the three representative devices as a function of the gate-voltage scan step, plotted as (a) drain current (*I*_D_) on a logarithmic scale and (b) the corresponding square root of the |*I*_D_|.

**Figure S21.** Apparent density-of-states (DOS) profiles for 0.1 (left) and 1 Å s^−1^ (right) devices extracted from photo-excited charge collection spectroscopy (PECCS) measurements.

**Figure S22.** Photo-induced |*I*_D_|^1/2^ curves during the PECCS measurement for the three representative devices. The insets show enlarged views of the initial negative *V*_th_ shift.

**Figure S23.** Temperature-dependent (a) field-effect mobility (*μ*_FE_), (b) subthreshold swing (*SS*) and (c) hysteresis (*ΔV*_th_) of the 0.1 and 10 Å s⁻¹ devices, extracted from transfer characteristics measured over 77–293 K.

**Figure S24.** Transfer characteristics of the devices fabricated with varying deposition rates and film thicknesses.

References

1.	S. Gossé, "Tellurium Oxides: Thermodynamics and Phase Relations in the Te–O System*",* *Journal of Phase Equilibria and Diffusion* (**2025):** 46, 20.

2.	M. Aspiala, D. Sukhomlinov, P. Taskinen, "Standard Gibbs Energy of Formation of Tellurium Dioxide Measurement by a Solid-Oxide Electrolyte EMF Technique*",* *Thermochimica Acta* (**2013):** 573, 95.

3.	S. Balakrishnan, R. Pankajavalli, K. Ananthasivan, et al., "Thermodynamic Stability of Sm_2_TeO_6_*",* *Thermochimica Acta* (**2008):** 467, 80.

4.	T. S. L. Narasimhan, M. S. Baba, R. Viswanathan, "Vaporization Behavior of TeO_2_(s): A Quantitative Vaporization—Knudsen Effusion Mass Spectrometric Study*",* *Thermochimica Acta* (**2005):** 427, 137.

5.	V. V. Trinadh, P. Manikandan, A. Jain, et al., "Vaporisation Studies on Sn-Te-O System by Knudsen Effusion Mass Spectrometry: Thermodynamic Properties over (SnO_2_+SnTe_3_O_8_) Biphasic Region*",* *Calphad* (**2019):** 64, 347.
